# Patterns of Leisure-Time Physical Activity Participation in a British Birth Cohort at Early Old Age

**DOI:** 10.1371/journal.pone.0098901

**Published:** 2014-06-09

**Authors:** Kathryn R. Martin, Rachel Cooper, Tamara B. Harris, Soren Brage, Rebecca Hardy, Diana Kuh, on behalf of the NSHD scientific and data collection team

**Affiliations:** 1 Epidemiology Group, School of Medicine and Dentistry, University of Aberdeen, Aberdeen, United Kingdom; 2 Medical Research Council Unit for Lifelong Health and Ageing at University College London, London, United Kingdom; 3 Laboratory of Epidemiology and Population Sciences, National Institute of Aging, National Institutes of Health, Bethesda, Maryland United States of America; 4 Medical Research Council Epidemiology Unit, School of Clinical Medicine, Institute of Metabolic Science, University of Cambridge, Cambridge, United Kingdom; Oregon Health & Science University, United States of America

## Abstract

Using data from a nationally representative British birth cohort we characterized the type and diversity of leisure-time physical activity that 2,188 participants (age 60–64 years) engaged in throughout the year by gender and obesity. Participants most commonly reported walking (71%), swimming (33%), floor exercises (24%) and cycling (15%). Sixty-two percent of participants reported ≥2 activities in the past year and 40% reported diversity on a regular basis. Regular engagement in different types of activity (cardio-respiratory, balance/flexibility and strength) was reported by 67%, 19% and 11% of participants, respectively. We found gender differences, as well as differences by obesity status, in the activities reported, the levels of activity diversity and activity type. Non-obese participants had greater activity diversity, and more often reported activities beneficial for cardio-respiratory health and balance/flexibility than obese participants. These findings may be used to inform the development of trials of physical activity interventions targeting older adults, and those older adults with high body mass index.

## Introduction

Leisure-time physical activity (LTPA) can be defined as volitional activity obtained through participation in sports, exercise and recreation at a moderate and/or vigorous intensity level, independent of other key physical activity (PA) domains, i.e., active transport (purposeful walking or cycling), domestic and occupational activity [1], [2]. In most high-income nations, modern technology and amenities have reduced the necessity for regular higher-level intensity activity via housework, physically demanding occupations, and active transport [3]. This is reflected in time-trend data showing declines in the average amount of time spent in domestic and work activity domains over recent decades [4], [5]; trends in active transport have been mixed, indicating both a decline [4] and an increase in this domain [6], [7]. Unlike other domains, time spent in LTPA has either remained stable [7], [8] or increased in several countries [2], [6], including the United Kingdom (UK) [9], [10]. Despite this population-level increase in LTPA, the proportion of UK adults meeting the minimum recommended PA guidelines (≥30 minutes of moderate or vigorous intensity PA on ≥5 days a week) was low in the Health Survey for England 2008 [10], [11]. Among older (≥65 years) UK adults, only 16% of men and 12% of women met the guidelines [10], [11], with exercise and fitness activities constituting only a small proportion of time spent in moderate-to-vigorous PA [11]. Data from Sport England's 2012/13 Active People Survey suggest that, of English adults aged 55–64 and ≥65 years, 61.6% and 73.9%, respectively, do not play any sports [12]. However, to achieve recommended levels of PA, LTPA is thought to be the domain most amenable to intervention especially in later life because other domains (e.g. occupational activity) are unlikely to be viable targets due to factors including retirement. Medical and public health professionals, as well as policymakers, have therefore focused intently on increasing the levels of activity obtained through LTPA at all ages, including older adults, in efforts to improve population health [13], [14].

Physical inactivity is associated with increased risk of numerous chronic conditions, cognitive decline and physical disability, as well as premature mortality [15]–[19]. Given the health benefits of PA, previous research has explored and identified numerous real and/or perceived personal and environmental barriers to initiating and maintaining PA [20], [21]. In older adults, barriers to PA can include current health status, availability of social support, and access to safe and affordable resources [21]–[24]. Additionally, a narrower range of different types of LTPAs may be acceptable and/or feasible in older adults because of increasing chronic disease burden and perceived and real injury risk. To help health promotion programs to be successful in improving levels of LTPA, it may also be useful to examine what sports and exercises adults in early old age organically engage in during their leisure-time, identifying the types and diversity of activity that are most acceptable and therefore potentially amenable to intervention.

Only a few nationally representative studies have examined the type and diversity of LTPA self-reported by community-dwelling older adults. Walking for exercise was the most commonly reported LTPA among older Americans (≥60 years) [25], Australians (≥65 years) [26], European Union citizens (≥55 years) [3], and English adults (≥65 years) [11]. After walking, activities such as cycling, golf, aerobics, swimming/water sports, and stretching were most often reported [3], [25]. Less than a third of older Australians reported two or more LTPA activities and only 2.6% reported engaging in a combination of aerobic, balance and flexibility exercises, as well as strength activities [26].

Health promotion programs also need to take account of how gender and obesity may influence type and diversity of LTPA. Studies of younger adults have highlighted notable gender differences in LTPA type, with men engaging in sports, jogging/running, cycling, and golf more often than women [27]–[29], however gender differences may change with increasing age. Previous research suggests that non-obese individuals are more likely to engage in LTPA than obese individuals [3], [29] but it is less clear whether type and diversity of activity are also influenced by obesity status in older age.

The aim of this study was to use data from the Medical Research Council (MRC) National Survey of Health and Development (NSHD) (1946 British Birth Cohort) to describe the types and diversity of leisure-time sports and exercise activities that adults aged 60–64 years report undertaking by gender and obesity status.

## Particpants And Methods

### Sample

The MRC NSHD is an ongoing birth cohort study of a socially-stratified sample of 5,362 males and females who have been followed up regularly since their birth in England, Scotland and Wales in 1946 [30], [31]. The most recent data collection took place between 2006 and 2010 when participants were aged 60–64 years. In 2006, the target sample consisted of 3,163 participants; 718 had previously died, 567 were living abroad, 594 had withdrawn from the study and 320 had been untraceable for more than 10years [31], [32]. Study members received postal questionnaires and were invited to attend a clinic visit or receive a nurse home visit. Information was obtained from the postal questionnaire and/or visits from 2,662 (84%) of the target sample. Of these, 2,224 participants (1,062 men and 1,162 women) completed a postal pre-assessment questionnaire prior to the clinic or home visit that included questions on physical activity. Data from 2,188 (1,046 men and 1,142 women) with no missing data on LTPA or body mass index (BMI) were used in the current analyses.

Ethical approval for the data collection at 60–64 years was obtained from the Central Manchester Local Research Ethics Committee and the Scotland A Research Ethics Committee. Written informed consent was obtained from study members at each stage of data collection. Bona fide researchers can apply to access the NSHD data via a standard application procedure (further details available at: http://www.nshd.mrc.ac.uk/data.aspx).

### Assessment of Type and Diversity of Leisure-Time Physical Activity

Physical activity was assessed using the EPIC-Norfolk Physical Activity Questionnaire (EPAQ2), which is a previously validated self-completed questionnaire that assesses activity in different domains of life – work, home and leisure [33]. In the current study, we used a modified version of this questionnaire which included the addition of two ‘free text boxes’ for participants to record other physical activities not in the pre-defined lists. LTPA was assessed by self-report of the frequency and duration of time spent in each of 30 pre-defined sports and exercise activities. Participants indicated the frequency of activity (choice of eight frequency categories ranging from ‘not done in last year’ to ‘six times a week/every day’) and gave the average time per episode in hours and minutes. We followed established methodology for data handling [27], [33], [34]. A duration variable (minutes/week) was derived for each leisure-time activity. For each of the activities, two variables were created: ‘report of any participation in the last year’ and ‘report of regular weekly participation of at least 30 minutes per episode.’ Ten activities were grouped to create five summary activities: swimming (leisure swimming and competitive swimming), running/jogging (jogging and competitive running), cycling (leisure cycling and racing cycling), aerobics (high-impact and other) and racquet sports (tennis and squash). In addition, we quantified the number of participants reporting zero, one, two, three, or four or more activities to assess level of activity diversity. Finally, we examined regular participation by ‘activity type’ categorized as: (1) cardio-respiratory, which included: walking for pleasure; back-packing/hill-walking; running/jogging; golf; swimming; cycling; aerobics; rowing; racquet sports; netball/volleyball/basketball; football; cricket; horse-riding; ice-skating; winter-sports (e.g. skiing); (2) activities beneficial for balance and flexibility, which included: martial arts; dancing; floor exercises; (3) activities beneficial for strength, which included: exercises with weights; conditioning exercises; and (4) all combinations, defined as a combination of cardio-respiratory, balance and flexibility and strength activity types [26].

### Covariates

Gender and body mass index (BMI) were explored as potential explanatory factors. Height and weight were measured by nurses using a standardized protocol; BMI was calculated (weight (kg)/height(m)^2^) and categorized as non-obese (<30 kg/m^2^) and obese (≥30 kg/m^2^).

### Statistical analysis

We examined the percent of participants reporting each sport and exercise activity in the past year and calculated the corresponding mean (SD) number of minutes per week among those who reported each activity. We formally tested whether differences in participation existed by gender for each activity reported in the last year and also on a regular basis (i.e. ≥30 min/week). We also tested whether participation in each of the activities differed by BMI status within gender. We then examined the difference between gender, as well as BMI status within gender, with regard to the number of different activities (i.e. diversity) and activity type (cardio-respiratory, balance and flexibility, and strength). Tests of difference were conducted using chi-squared and Fisher's exact tests; Ns, percent participation (%), mean minutes per week (SD), and 95% confidence intervals are reported and we list top 10 activities by gender and BMI status (stratified by gender). All statistical analyses were conducted using STATA v13.0 (STATA Corp. – College Station, Texas).

## Results

**Figure 1:**
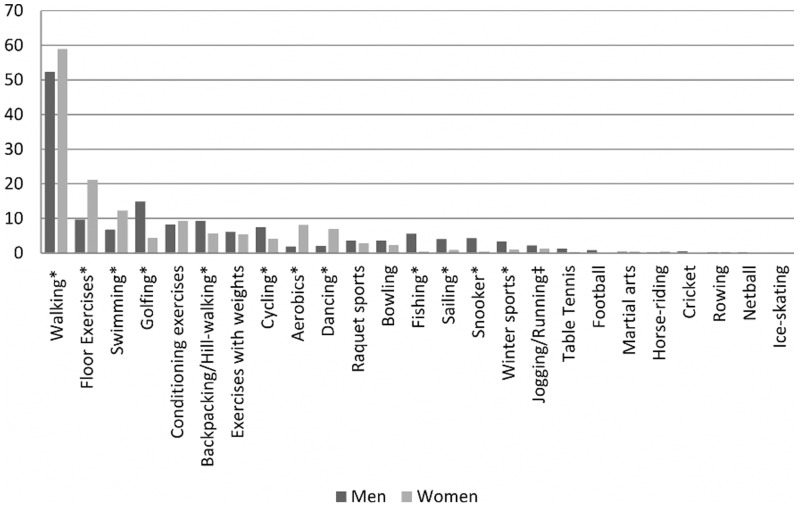
The percentage (%) of participants, (n^§^ = 2188) reporting sport and exercise activities on a regular basis^‡^, by gender. ^§^ Percentages reported are based on available n for each specific activity; ^‡^‘report of weekly participation in ‘x’ activity of minimum duration, 30 minutes per episode’. ^*^p<0.05; ^‡^p<0.10: test of gender difference. *Note*: No difference by gender existed for exercises with weights and conditioning exercises either in the last year or report of regular participation; N's were not robust to examine football, horse-riding, cricket, ice-skating, martial arts, and netball by gender due to low number of participants reporting these activities. Activity order was determined by the most frequent activity in both genders combined. Top ten most frequently reported activities by gender (where ‘1’ indicates most frequently reported activity): *Men*: 1) walking for pleasure, 2) golfing, 3) floor exercises, 4) backpacking/hill-walking, 5) conditioning exercises, 6) cycling, 7) swimming, 8) exercises with weights, 9) fishing and 10) snooker. *Women*: 1) walking for pleasure, 2) floor exercises, 3) swimming, 4) conditioning exercises, 5) aerobics, 6) dancing, 7) backpacking/hill-walking, 8) exercises with weights, 9) golfing and 10) cycling.

The percentage of participants reporting participation in each sport and exercise activity in the past year, as well as the mean number of minutes per week participants reported undertaking an activity are given in Table 1. Overall, 71% of participants reported walking for pleasure in the past year for an average of 192.7 minutes per week. Other activities were less frequently reported; the top 10 most commonly reported activities in the last year were swimming (33%), floor exercises (24%), conditioning exercises (16%), cycling (15%), backpacking/hill-walking (14%), dancing (14%), golfing (12%), exercises with weights (11%), and aerobics (8%). Walking for pleasure was also the most common activity reported on a regular basis (≥30 minutes per week), as it was reported by 59% of women and 52% of men (test of gender difference, *p* = 0.002). While similarities existed between men and women with regard to the top 10 activities reported on a regular basis, gender differences were observed in the percentage of individuals reporting each given activity. Briefly, women more often participated in walking for pleasure, swimming, aerobics, floor exercises, and dancing on a regular basis (Figure 1), whereas men more often engaged in backpacking/hill-walking, jogging and running, cycling and golfing.

**Table 1:** The percentage (%) of participants reporting each sport and exercise activity in the past year and mean (SD) time (minutes per week) spent in each activity.

		% reporting participation	Mean minutes
	N*	in past year (N)	per week (SD)‡
Walking for pleasure	2187	71 (1555)	192.7 (300.5)
Swimming^a^	2187	33 (725)	33.3 (61.1)
Floor exercises	2176	24 (524)	66.3 (84.7)
Conditioning exercises	2176	16 (342)	53.7 (57.1)
Cycling^b^	2187	15 (328)	70.4 (158.2)
Backpacking/Hill-walking	2187	14 (310)	85.0 (167.6)
Dancing	2170	14 (296)	56.6 (112.3)
Golfing	2175	12 (266)	309.1 (298.5)
Exercises with weights	2176	11 (236)	53.4 (77.8)
Aerobics^c^	2176	8 (163)	66.1 (76.7)
Bowling	2175	7 (161)	100.6 (166.6)
Snooker	2175	6 (130)	70.5 (115.8)
Racquet sports^d^	2175	5 (113)	100.1 (123.8)
Fishing	2170	4 (92)	171.2 (65.2)
Sailing	2170	4 (85)	160.3 (409.1)
Jogging/running^e^	2187	3 (71)	58.1 (69.8)
Table Tennis	2175	3 (59)	36.1 (75.2)
Winter sports (e.g., skiing)◊	2170	3 (57)	444.9 (790.1)
Rowing	2170	1 (26)	27.1 (43.4)
Football◊	2175	<1 (20)	43.1 (42.7)
Horse-riding	2170	<1 (17)	169.6 (205.5)
Ice-skating	2170	<1 (14)	20.4 (37.5)
Cricket◊	2175	<1 (13)	104.8 (137.7)
Martial arts	2170	<1 (9)	113.3 (66.7)
Netball, volleyball, basketball	2170	<1 (4)	22.7 (17.7)

*N varies due to missing data for specific activities.

‡ Represents mean time spent in activity among those reporting activity participation in past year.

a : Combined swimming leisurely and competitive swimming; ^b^: Combined cycling recreational and competitive cycling; ^c^: Combined high-impact aerobics and other aerobics; ^d^Combined tennis and squash; ^e^Combined jogging and competitive running.

◊ Values represent time spent in activity during the season.

**Figure 2:**
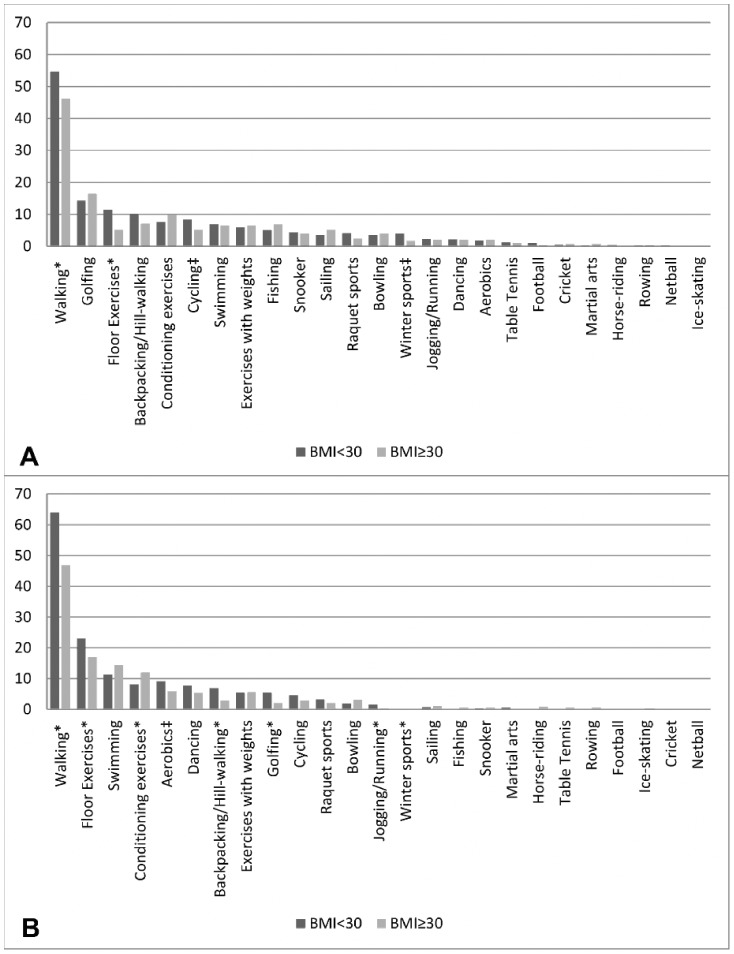
The percentage (%) of men (n^§^ = 1046) and women (n^§^ = 1142) reporting sport and exercise activities on a regular basis^‡^, by obesity status. ^§^ Percentages reported are based on available n for each specific activity;^ ‡^‘report of weekly participation in ‘x’ activity of minimum duration, 30 minutes per episode’. ^*^p<0.05; ^‡^p<0.10: test of difference by obesity status. (A) Activity order was determined by the most frequent activity among men. Top ten most frequently reported activities among men (where ‘1’ indicates most frequently reported activity): *BMI<30 kg/m^2^*: 1) walking for pleasure, 2) golfing, 3) floor exercises, 4) backpacking/hill-walking, 5) conditioning exercises, 6) cycling, 7) swimming, 8) fishing, 9) exercises with weights and 10) snooker. *BMI≥30 kg/m^2^*: 1) walking for pleasure, 2) golfing, 3) conditioning exercises, 4) backpacking/hill-walking, 5) fishing, 6) exercises with weights, 7) swimming, 8) floor exercises, 9) sailing and 10) cycling. (B) Activity order was determined by the most frequent activity among women. Top ten most frequently reported activities among women (where ‘1’ indicates most frequently reported activity): *BMI<30 kg/m^2^*: 1) walking for pleasure, 2) floor exercises, 3) swimming, 4) aerobics, 5) conditioning exercise, 6) dancing,7) backpacking/hill-walking, 8) exercises with weights, 9) golfing and 10) cycling. *BMI≥30 kg/m^2^*: 1) walking for pleasure, 2) floor exercises, 3) swimming, 4) conditioning exercise, 5) aerobics, 6) exercises with weights, 7) dancing, 8) bowling, 9) backpacking/hill-walking and 10) cycling.

Among men (Figure 2A), the top 10 regular reported activities were similar by obesity status, however differences were observed in the percentage of individuals reporting each given activity. A greater percentage of non-obese men regularly engaged in walking for pleasure (55% vs. 46%, *p* = 0.02) and floor exercises (12% vs. 5%, *p* = 0.002) than obese men. A trend for non-obese men to more often report cycling (8% vs. 5%, *p* = 0.08) than obese men was also observed. Similar to men, among women the top 10 regular activities were comparable by obesity status but again differences were observed in the reported percentages. Among women (Figure 2B), those who were non-obese more often reported regularly walking for pleasure (64% vs.47%, *p*<0.001), engaging in floor exercises (23% vs. 17%, *p* = 0.02), as well as backpacking/hill-walking (7% vs. 3%, p = 0.01), and golfing (5% vs 2%, *p* = 0.01) when compared to obese women. However, obese women more frequently reported taking part in conditioning exercises (12% vs. 8%, p = 0.04) than non-obese women.

Overall, 16% of participants reported no engagement in any of the activities at least once within the past year, while approximately 21% engaged in one, 19% in two, 15% in three and 28% in four or more activities; the distribution across groups was comparable by gender (*p* = 0.2) (Table 2). When restricting analyses to activities participants engaged in on a regular basis, there was less activity diversity; nearly 27% of participants reported no regular engagement in any of the activities, 34% reported one activity, 20% reported two, 11% reported three, and just 9% of participants reported regular engagement in four or more different activities. Diversity of regular activity was comparable by gender (*p* = 0.9). The majority of activity was classified as cardio-respiratory and the percentage engaging in cardio-respiratory activity was comparable by gender, both at least once throughout the year (*p* = 0.2) and on a regular basis (*p* = 0.3). However, when compared to men, women were more likely to engage in activities beneficial for balance and flexibility at least once throughout the year and on a regular basis (both *p*<0.001). A lower percentage of men and women engaged in all combinations of activity on a regular basis than at any point throughout the year. When compared to men, women were more likely to engage in all combinations of activity at least once throughout the year (*p* = 0.02) and on a regular basis (*p* = 0.04).

**Table 2:** Diversity in leisure-time physical activity and participation in different activity-types, % (CI), by gender and obesity status.

		Men			Women	
	Total (n = 1,046)	BMI <30 (n = 754)	BMI ≥30 (n = 292)	Total (n = 1,142)	BMI <30 (n = 801)	BMI ≥30 (n = 341)
	% (CI)	% (CI)	% (CI)	% (CI)	% (CI)	% (CI)
Diversity within year□						
0 activities	16.1 (13.9, 18.4)	13.9 (11.5, 16.6)*	21.6 (17.0, 26.7)	15.9 (13.8, 18.1)	12.9 (10.6, 15.4)*	22.9 (18.5, 27.7)
1 activity	21.6 (19.1, 24.2)	22.7 (19.7, 25.8)	18.8 (14.5, 23.8)	21.1 (19.0, 23.8)	20.9 (18.2, 24.0)	22.3 (17.9, 27.1)
2 activities	18.8 (16.5, 21.3)	18.6 (15.9, 21.5)	19.5 (15.1, 24.5)	20.4 (17.8, 22.5)	20.1 (17.4, 23.0)	19.9 (15.8, 24.6)
3 activities	14.0 (12.0, 16.3)	14.7 (12.3, 17.5)	12.3 (8.8, 16.7)	16.0 (14.8, 19.2)	18.0 (15.4, 20.8)	14.3 (10.8, 18.5)
4+ activities	29.5 (26.7, 32.3)	30.1 (26.8, 33.5)	27.7 (22.7, 33.3)	26.6 (23.3, 28.5)	28.1 (25.0, 31.3)	20.5 (16.4, 25.2)
Diversity on a regular basis¥						
0 activities	26.7 (24.0, 29.5)	24.8 (21.8, 28.0)	31.5 (26.2, 37.2)	26.4 (23.8, 29.0)	22.2 (19.4, 25.3)*	36.1 (31.0, 41.4)
1 activity	33.3 (30.4, 36.2)	33.8 (30.4, 37.3)	31.8 (26.5, 37.5)	34.5 (31.7, 37.3)	35.4 (32.0, 38.8)	32.6 (27.6, 37.8)
2 activities	19.6 (17.2, 22.1)	20.2 (17.4, 23.2)	18.2 (13.9, 23.1)	19.5 (17.3, 21.9)	21.8 (19.0, 24.9)	14.1 (10.6, 18.2)
3 activities	10.7 (8.9, 12.7)	10.9 (8.7, 13.3)	10.3 (7.0, 14.3)	11.1 (9.4, 13.1)	11.5 (9.4, 13.9)	10.3 (7.3, 14.0)
4+ activities	9.8 (8.0, 11.7)	10.3 (8.3, 12.7)	8.2 (5.3, 12.0)	8.5 (6.9, 10.3)	9.1 (7.2, 11.3)	7.0 (4.6, 10.3)
Activity-type within year□						
Cardio-respiratory^a^	77.8 (75.2, 80.3)	79.8 (76.8, 82.6)*	72.6 (67.1, 77.6)	80.3 (77.9, 82.6)	83.6 (80.9, 86.1)*	72.4 (67.4, 77.1)
Balance and flexibility^b^	23.9 (21.3, 26.6)*	25.6 (22.5, 28.9)*	19.5 (15.1, 24.5)	39.6 (36.7, 42.5)	41.7 (38.3, 45.2)*	34.6 (29.6, 39.9)
Strength^c^	17.5 (15.2, 19.9)	17.1 (14.5, 20.0)	18.5 (14.2, 23.4)	19.4 (17.2, 21.9)	19.1 (16.4, 22.0)	20.2 (16.1, 24.9)
All combinations^d^	10.5 (8.7, 12.5)*	10.6 (8.5, 13.0)	10.3 (7.0, 14.3)	13.9 (12.0, 16.1)	14.1 (11.8, 16.7)	13.5 (10.0, 17.6)
Activity-type on a regular						
basis¥						
Cardio-respiratory^a^	65.8 (62.8, 68.6)	67.9 (64.4, 71.2)*	60.3 (54.4, 65.9)	67.9 (65.1, 70.6)	72.5 (69.3, 75.6)*	56.9 (51.4, 62.2)
Balance and flexibility^b^	11.3 (9.4, 13.4)*	12.9 (10.6, 15.5)*	7.2 (4.5, 10.8)	25.7 (23.2, 28.4)	27.8 (24.8, 31.1)*	20.8 (16.6, 25.5)
Strength^c^	10.8 (9.0, 12.8)	10.3 (8.3, 12.7)	12.0 (8.5, 16.3)	11.8 (10.0, 13.8)	10.7 (8.7, 13.1)	12.9 (9.5, 16.9)
All combinations^d^	3.8 (2.7, 5.2)*	4.1 (2.8, 5.8)	3.1 (1.4, 5.8)	5.7 (4.4, 7.2)	5.5 (4.0, 7.3)	6.2 (3.9, 9.3)

Note: Values for football, cricket and winter sports (e.g., skiing) represented time spent in activity during the season.

□ Any report of activity (activities) in last year.

¥ Report of average participation in activity (activities) for 30+ min per week; note that values used for skiing, winter sport football and cricket represented time spent in season.

a cardio-respiratory, which included: walking for pleasure; backpacking/hill-walking; running/jogging; golfing; swimming; cycling; aerobics; rowing; racquet sports; netball/volleyball/basketball; football; cricket; horse-riding; ice-skating; winter-sports (e.g., skiing); ^b^Martial arts, dancing, floor exercises; ^c^Exercises with weights, conditioning exercises; ^d^Combination of Cardio-respiratory, Balance and flexibility and Strength.

*Indicates *p*<0.05 for chi-squared test of association by BMI status within gender.

BMI was associated with both activity diversity and activity type for both men and women (Table 2). A greater percentage of non-obese men reported engaging in four or more activities, while a greater percentage of obese men reported no participation in any of the activities at any point throughout the year (*p = *0.03). Diversity was not significantly associated with obesity status among men when examining activity on a regular basis (*p* = 0.3), though trends remained. In women, however, diversity of activity was associated with obesity status both throughout the year and on a regular basis (both *p*<0.001). When examining the activity type reported, a greater percentage of non-obese men and women reported cardio-respiratory activity at some point throughout the year (*p* = 0.01 and *p*<0.001, respectively) and on a regular basis (*p* = 0.02 and *p*<0.001, respectively), than did obese participants. In addition, a greater percentage of non-obese men also reported activities beneficial for balance and flexibility at some point throughout the year (*p* = 0.04), as well as on a regular basis (*p* = 0.01). This was also observed among women throughout the year (*p* = 0.03), as well as on a regular basis (*p* = 0.01). No difference was observed by BMI status for participation in activities beneficial for strength or all combinations of activities among men and women (all *p*>0.05).

## Discussion

Using data from a nationally representative study, we have characterized the types and diversity of LTPA that British adults in early old age reported undertaking in the past year. We found gender differences, as well as differences by obesity status, in the activities reported, the levels of activity diversity and activity types. Walking for pleasure was the most commonly reported LTPA among both men and women, and these findings are consistent with the literature [3], [11], [25], [26]. In addition to walking, we found that 62% of participants reported diversity (i.e. ≥2 unique activities) of leisure-time activities at least once throughout the year, but only 40% reported doing so on a regular basis. Levels of participation diversity were higher in this study than previously reported in a sample of older Australians (≥65years) [26].

There were gender differences found in the activities reported. Among the list of the top 10 activities undertaken on a regular basis, a greater percentage of men reported golfing, cycling, and backpacking/hill-walking, whereas more women reported walking for pleasure, floor exercises, aerobics, dancing and swimming. The activities men more often reported engaging in are of greater intensity and have higher associated metabolic equivalent (MET) values [35], [36] yet high-impact team activities (e.g. football) that are often popular in earlier adulthood, especially among men [28], are noticeably absent from the top 10 list in this study. Indeed, football was one of the most frequently reported activities among men in the NSHD at age 36 years [37]. Swimming (in both sexes) and golf (in men) were among the most frequently reported activities at both ages [37], suggesting that such lower intensity activities may be more likely to be maintained with increasing age than more vigorous activities such as football and running. That more of the activities frequently reported among women at age 36 years (e.g. swimming and dancing) were also in the top 10 most frequently reported activities at age 60–64 years [37], suggests that greater changes in activity type may occur among men than women with increasing age.

Study results also suggest that activity diversity and activity type varied by obesity status among men and women. Non-obese women reported greater activity diversity on a regular basis than obese women, though this relationship was not observed among men. These results are in line with findings from a Canadian study that found the prevalence of physical inactivity increased with increasing BMI among women, but not in men and that the largest difference between BMI status occurred among the oldest age group (50–64 years) [38]. In the current study, lower BMI was associated with more regular participation in cardio-respiratory activity, as well as balance and flexibility activity for both men and women. To our knowledge, this study is the first to examine the relationship between leisure-time sport and exercise activity, gender and BMI in this way. Despite different PA measurement and operationalization (e.g., leisure METS), our findings are also supported by prior research suggesting an association between higher levels of recreational activity and lower BMI, as well as no association for vigorous active recreation (time spent in any activity of ≥5METS) among men by BMI status [29].

Although walking is often recommended to adults, especially those overweight and/or older, as a way to increase PA levels through low-impact activity, our study found that regular weekly walking for pleasure varied by BMI status among both men and women. This difference was not observed among another low-impact activity, swimming, which is commonly recommended to older and/or overweight populations as a low weight-bearing, joint-friendly activity [39], [40]. While the difference in reports observed by BMI status may reflect lower BMI as a result of regular walking for pleasure during one's lifetime or adulthood, our findings may also indicate that obese individuals face additional barriers to walking for pleasure (e.g. pain, physical disability, embarrassment) that go beyond, but are also related to body size and weight [41]. Indeed, findings from the EPIC-Norfolk cohort suggest that weight status and weight gain are associated with both contemporaneous levels and subsequent changes in physical inactivity [42]. Combined with findings from the current study, this may suggest that the benefits of walking should be promoted within a context that acknowledges personal, social, and/or environmental barriers among older adults with higher BMI, so as to increase engagement in walking for pleasure as a LTPA and reduce the observed difference by BMI status.

Previous research has found that, in contrast to domestic activities, leisure pursuits, outdoor pursuits, sports (team and non-team), and exercise and fitness contributed very little towards the proportion of total time per week spent in moderate-to-vigorous physical activity among English adults aged 65 years and older [11]. We extend these observations through our examination of diversity and activity type in the current study. While fewer than 10 percent of older adults reported high activity diversity, i.e. four or more different leisure sport and exercise activities on a regular basis, nearly a third were found to have high diversity throughout the year. Seasonality of activity participation may play a role in the finding of higher diversity when considering activity undertaken at any point throughout the year, as some activities can only be undertaken in specific seasons and better conditions exist for many activities (e.g., reduced access/transportation barriers, increased daylight, fairer weather conditions), especially those conducted outdoors, in spring and summer. However, these findings do suggest that older adults have varied activity interests and that there may be great potential for increasing the time older adults spend in sport and exercise activities by encouraging regular participation in a variety of diverse activities. That nearly 60 percent of participants reported either none or participation in just one of the activities on a regular basis represents an opportunity for public health practitioners and clinicians to discuss and encourage older adults to increase their PA through a varied and diverse routine of sport and exercise activities. Engagement in a greater number of different activities may be a strategy that results in greater time spent in overall activity, especially for older adults who are either overweight or obese.

Our study has several strengths. It is a nationally representative sample of adults in early old age, with good characterization of LTPA as measured by a validated questionnaire, the EPAQ2. However, some limitations should be noted. In general, self-report PA, as measured by questionnaire, is noted to suffer from recall bias, especially among older adults [43]–[45]. For example, participants may have difficulty remembering whether or not they engaged in a particular activity in the past, as well as the average length of time per episode. Self-report PA is also prone to social desirability bias, as participants might feel compelled to over-report activity levels knowing the benefits of PA on health. However, self-reported questionnaires are currently the best method for ascertaining information on participation in different types of activity, as these parameters of PA are not yet easily captured by objective monitoring. Further, we purposefully chose to focus on sport and exercise activities that are of higher intensity (moderate-to-vigorous) partly because they are reported with greater reliability and validity than lower intensity (light) activities [46], [47]. Additionally, higher intensity activities are often thought to enable older adults to meet the currently recommended PA levels. We were unable to explore reasons for choice of activity and explanations of non-participation, as we did not ask participants whether or not non-participation or low participation frequency was due to lack of interest, lack of opportunity/resources, or physical inability to engage in a specific activity. Nor did we ask participants if they independently engaged in an activity, if they were active with a group or someone in their social network, or even their preference for solo or group activity. Therefore, we suggest that these factors are explored in future research, as well as in advance of development/implementation of interventions to increase PA among older men and women.

## Conclusion

In conclusion, there is considerable variation in types and diversity of leisure sports and exercise activities in this population of British adults in early old age, and different patterns of activity were observed by gender and obesity status. While 40 percent engaged in two or more types of activity on a regular basis, the majority reported no diversity or regular participation in any of the activities queried. In general, walking for pleasure is the activity most often reported among older adults, perhaps due in part to its ease, convenience, lack of associated risk and inexpensive nature, as well as perhaps increased likelihood of exposure to an outdoor environment. Clinicians, public health professionals and researchers might capitalize on this knowledge when developing trials of PA interventions tailored and targeted to older men and women, and those with high BMI.

## References

[1] Moore SC, Patel AV, Matthews CE, Berrington de GA, Park Y, et al (2012) Leisure time physical activity of moderate to vigorous intensity and mortality: a large pooled cohort analysis. PLoS Med 9: e1001335. 23139642 10.1371/journal.pmed.1001335 PMC3491006

[2] Palacios-Cena D, Alonso-Blanco C, Jimenez-Garcia R, Hernandez-Barrera V, Carrasco-Garrido P, et al (2011) Time trends in leisure time physical activity and physical fitness in elderly people: 20 year follow-up of the Spanish population national health survey (1987–2006). BMC Public Health 11: 799 10.1186/1471-2458-11-799 21995560 PMC3206481

[3] Vaz de Almeida MD, Graca P, Afonso C, D'Amicis A, Lappalainen R, et al (1999) Physical activity levels and body weight in a nationally representative sample in the European Union. Public Health Nutr 2: 105–113. 10933630 10.1017/s1368980099000154

[4] Brownson RC, Boehmer TK, Luke DA (2005) Declining rates of physical activity in the United States: what are the contributors? Annu Rev Public Health 26: 421–443. 15760296 10.1146/annurev.publhealth.26.021304.144437

[5] Church TS, Thomas DM, Tudor-Locke C, Katzmarzyk PT, Earnest CP, et al (2011) Trends over 5 decades in U.S. occupation-related physical activity and their associations with obesity. PLoS One 6: e19657. 21647427 10.1371/journal.pone.0019657 PMC3102055

[6] Juneau CE, Potvin L (2010) Trends in leisure-, transport-, and work-related physical activity in Canada 1994–2005. Prev Med 51: 384–386. 20832417 10.1016/j.ypmed.2010.09.002

[7] Hallal PC, Knuth AG, Reis RS, Rombaldi AJ, Malta DC, et al (2011) Time trends of physical activity in Brazil (2006-2009). Rev Bras Epidemiol 14 Suppl 1: 53–60. 22002142 10.1590/s1415-790x2011000500006

[8] Moore LV, Harris CD, Carlson SA, Kruger J, Fulton JE (2012) Trends in no leisure-time physical activity—United States, 1988-2010. Res Q Exerc Sport 83: 587–591. 23367822 10.1080/02701367.2012.10599884

[9] Stamatakis E, Chaudhury M (2008) Temporal trends in adults' sports participation patterns in England between 1997 and 2006: the Health Survey for England. Br J Sports Med 42: 901–908. 18658250 10.1136/bjsm.2008.048082

[10] Craig R, Mindell J, Hirani V (2009) Health Survey for England 2008. Volume 1: Physical Activity and Fitness. Health Survey for England 8–395.

[11] Belanger M, Townsend N, Foster C (2011) Age-related differences in physical activity profiles of English adults. Prev Med 52: 247–249. 21338622 10.1016/j.ypmed.2011.02.008

[12] Sport England (2014) Active People Survey 2012/2013. Available: http://activepeople.sportengland.org/Querty. Accessed 2014 Mar 05

[13] Department of Health PAHIaP (2011) Start Active, Stay Active: a report on physical activity for health from the four home countries' Chief Medical Officers. Available: http://www.dh.gov.uk/en/Publicationsandstatistics/Publications/PublicationsPolicyAndGuidance/DH_128209.

[14] NICE (2013) Physical activity: brief advice for adults in primary care. Public Health Guidance 44.

[15] Warburton DE, Nicol CW, Bredin SS (2006) Health benefit of physical activity: the evidence. CMAJ 174: 801–809. 16534088 10.1503/cmaj.051351 PMC1402378

[16] Paterson DH, Warburton DE (2010) Physical activity and functional limitations in older adults: a systematic review related to Canada's Physical Activity Guidelines. Int J Behav Nutr Phys Act 7: 38 10.1186/1479-5868-7-38 20459782 PMC2882898

[17] Boyle PA, Buchman AS, Wilson RS, Bienias JL, Bennett DA (2007) Physical activity is associated with incident disability in community-based older persons. J Am Geriatr Soc 55: 195–201. 17302655 10.1111/j.1532-5415.2007.01038.x

[18] Xue QL, Bandeen-Roche K, Mielenz TJ, Seplaki CL, Szanton SL, et al (2012) Patterns of 12-year change in physical activity levels in community-dwelling older women: can modest levels of physical activity help older women live longer? Am J Epidemiol 176: 534–543. 22935515 10.1093/aje/kws125 PMC3530350

[19] Andersen LB, Schnohr P, Schroll M, Hein HO (2000) All-cause mortality associated with physical activity during leisure time, work, sports, and cycling to work. Arch Intern Med 160: 1621–1628. 10847255 10.1001/archinte.160.11.1621

[20] Wendel-Vos W, Droomers M, Kremers S, Brug J, van LF (2007) Potential environmental determinants of physical activity in adults: a systematic review. Obes Rev 8: 425–440. 17716300 10.1111/j.1467-789X.2007.00370.x

[21] Chipperfield JG, Newall NE, Chuchmach LP, Swift AU, Haynes TL (2008) Differential determinants of men's and women's everyday physical activity in later life. J Gerontol B Psychol Sci Soc Sci 63: S211–S218. 18689770 10.1093/geronb/63.4.s211 PMC3874240

[22] Moschny A, Platen P, Klaassen-Mielke R, Trampisch U, Hinrichs T (2011) Barriers to physical activity in older adults in Germany: a cross-sectional study. Int J Behav Nutr Phys Act 8: 121 10.1186/1479-5868-8-121 22047024 PMC3225299

[23] Salmon J, Owen N, Crawford D, Bauman A, Sallis JF (2003) Physical activity and sedentary behavior: a population-based study of barriers, enjoyment, and preference. Health Psychol 22: 178–188. 12683738 10.1037//0278-6133.22.2.178

[24] Finch C, Owen N, Price R (2001) Current injury or disability as a barrier to being more physically active. Med Sci Sports Exerc 33: 778–782. 11323548 10.1097/00005768-200105000-00016

[25] Hughes JP, McDowell MA, Brody DJ (2008) Leisure-time physical activity among US adults 60 or more years of age: results from NHANES 1999-2004. J Phys Act Health 5: 347–358. 18579914 10.1123/jpah.5.3.347

[26] Merom D, Carmen C, Kamalesh V, Adrian B (2012) How diverse was the leisure time physical activity of older Australians over the past decade? J Sci Med Sport 15: 213–219. 22197582 10.1016/j.jsams.2011.10.009

[27] Parsons TJ, Thomas C, Power C (2009) Estimated activity patterns in British 45 year olds: cross-sectional findings from the 1958 British birth cohort. Eur J Clin Nutr 63: 978–985. 19223916 10.1038/ejcn.2009.6

[28] Deaner RO, Geary DC, Puts DA, Ham SA, Kruger J, et al (2012) A sex difference in the predisposition for physical competition: males play sports much more than females even in the contemporary U.S. PLoS One 7: e49168. 23155459 10.1371/journal.pone.0049168 PMC3498324

[29] Livingstone MB, Robson PJ, McCarthy S, Kiely M, Harrington K, et al (2001) Physical activity patterns in a nationally representative sample of adults in Ireland. Public Health Nutr 4: 1107–1116. 11820924 10.1079/phn2001192

[30] Wadsworth M, Kuh D, Richards M, Hardy R (2006) Cohort Profile: The 1946 National Birth Cohort (MRC National Survey of Health and Development). Int J Epidemiol 35: 49–54. 16204333 10.1093/ije/dyi201

[31] Kuh D, Pierce M, Adams J, Deanfield J, Ekelund U, et al (2011) Cohort profile: updating the cohort profile for the MRC National Survey of Health and Development: a new clinic-based data collection for ageing research. Int J Epidemiol 40: e1–e9. 21345808 10.1093/ije/dyq231 PMC3043283

[32] Stafford M, Black S, Shah I, Hardy R, Pierce M, et al (2013) Using a birth cohort to study ageing: representativeness and response rates in the National Survey of Health and Development. Eur J Ageing 10: 145–157. 23637643 10.1007/s10433-013-0258-8 PMC3637651

[33] Wareham NJ, Jakes RW, Rennie KL, Mitchell J, Hennings S, et al (2002) Validity and repeatability of the EPIC-Norfolk Physical Activity Questionnaire. Int J Epidemiol 31: 168–174. 11914316 10.1093/ije/31.1.168

[34] Espana-Romero V, Golubic R, Martin KR, Hardy R, Ekelund U, et al (2014) Comparison of the EPIC Physical Activity Questionnaire with Combined Heart Rate and Movement Sensing in a Nationally Representative Sample of Older British Adults. PLoS One 9: e87085. 24516543 10.1371/journal.pone.0087085 PMC3916297

[35] Ainsworth BE, Haskell WL, Leon AS, Jacobs DR Jr, Montoye HJ, et al (1993) Compendium of physical activities: classification of energy costs of human physical activities. Med Sci Sports Exerc 25: 71–80. 8292105 10.1249/00005768-199301000-00011

[36] Ainsworth BE, Haskell WL, Whitt MC, Irwin ML, Swartz AM, et al (2000) Compendium of physical activities: an update of activity codes and MET intensities. Med Sci Sports Exerc 32: S498–S504. 10993420 10.1097/00005768-200009001-00009

[37] Kuh DJ, Cooper C (1992) Physical activity at 36 years: patterns and childhood predictors in a longitudinal study. J Epidemiol Community Health 46: 114–119. 1583424 10.1136/jech.46.2.114 PMC1059517

[38] Chen Y, Mao Y (2006) Obesity and leisure time physical activity among Canadians. Prev Med 42: 261–265. 16476475 10.1016/j.ypmed.2006.01.006

[39] Waumsley J, Mutrie N (2011) Physical Activity and Exercise Psychology: Our role in healthy weight management for adults. Obesity in the UK: A psychological perspective 5–15.

[40] Young A, Dinan S (1994) ABC of sports medicine. Fitness for older people. BMJ 309: 331–334. 8086874 10.1136/bmj.309.6950.331 PMC2540892

[41] Ball K, Crawford D, Owen N (2000) Too fat to exercise? Obesity as a barrier to physical activity. Aust N Z J Public Health 24: 331–333. 10937415 10.1111/j.1467-842x.2000.tb01579.x

[42] Golubic R, Ekelund U, Wijndaele K, Luben R, Khaw KT, et al (2013) Rate of weight gain predicts change in physical activity levels: a longitudinal analysis of the EPIC-Norfolk cohort. Int J Obes (Lond) 37: 404–409. 22531093 10.1038/ijo.2012.58 PMC3635037

[43] Prince SA, Adamo KB, Hamel ME, Hardt J, Connor GS, et al (2008) A comparison of direct versus self-report measures for assessing physical activity in adults: a systematic review. Int J Behav Nutr Phys Act 5: 56 10.1186/1479-5868-5-56 18990237 PMC2588639

[44] Loney T, Standage M, Thompson D, Sebire SJ, Cumming S (2011) Self-report vs. objectively assessed physical activity: which is right for public health? J Phys Act Health 8: 62–70. 21297186 10.1123/jpah.8.1.62

[45] Colbert LH, Matthews CE, Havighurst TC, Kim K, Schoeller DA (2011) Comparative validity of physical activity measures in older adults. Med Sci Sports Exerc 43: 867–876. 20881882 10.1249/MSS.0b013e3181fc7162 PMC3303696

[46] Bonnefoy M, Normand S, Pachiaudi C, Lacour JR, Laville M, et al (2001) Simultaneous validation of ten physical activity questionnaires in older men: a doubly labeled water study. J Am Geriatr Soc 49: 28–35. 11207839 10.1046/j.1532-5415.2001.49006.x

[47] Lee IM, Shiroma EJ (2014) Using accelerometers to measure physical activity in large-scale epidemiological studies: issues and challenges. Br J Sports Med 48: 197–201. 24297837 10.1136/bjsports-2013-093154 PMC3947179

